# Populism and Protest

**DOI:** 10.3389/fsoc.2020.619235

**Published:** 2021-02-10

**Authors:** Nancy J. Hirschmann

**Affiliations:** Department of Political Science, University of Pennsylvania, Philadelphia, PA, United States

**Keywords:** populism, protest, feminism, black lives matter movement, anti-racism campaign, violence, masculinity

## Abstract

The essay considers populism in the present moment in relation to Black Lives Matter as a popular protest movement. Popular protest movements demand that government change; populism in the present moment seeks to act extra-governmentally, and to this end relies on violence in the face of peace protest movement. This violence demonstrates the white patriarchalism of contemporary populism. I argue that peaceful, popular protest is an important tool to resist white patriarchal populist authoritarianism.

## Introduction

George Floyd’s murder in May 2020 by a white police officer who kneeled on his neck for more than 8 min, in apparent mockery of his victim’s suffering, triggered a series of protests throughout the United States and the world. Though the Black Lives Matter (BLM) movement began in 2013 after the murder of Trayvon Martin whose white murderer was acquitted by a jury in Florida, and gained strength in response to police killings in Ferguson, MO and New York City, it was George Floyd’s murder that triggered a series of protests over killings in Kenosha, WI, Portland, OR, and Philadelphia as thousands more people of all races joined in sustained protest marches through the pandemic summer of 2020. Because of these numbers, as well as the widespread location of the protests, including suburban towns, some might consider BLM protest activity “populist”— marches and candle light vigils, not only in the United States but throughout the world; the wall of moms and leaf-blower dads in Portland, Oregon. In light of these it’s easy to think that populism must be a good thing; that’s what democracy is all about, government by the people and for the people, right? *Vox populi, vox Dei.*

Yet many on the left are unhappy with populism, which gave us Donald Trump, Brexit, and the Italian League. While educated Americans like to fantasize that Europeans are more sensible and civil about their politics—universal health care is accepted as a norm, not as a radical evil that will destroy society (despite the popularity of Medicare)— the European anti-immigration rhetoric of hate, exclusion, and domination signaled a shift in the political climate that seemed to lead directly to Trump in the United States, layered onto its history of racism, misogyny, and xenophobia.

Populism in the United States is generally traced to the People’s Party in the late 19th century, a progressive movement that advocated women’s suffrage and the rights of black farmers, some trace it to darker roots, in the election of Andrew Jackson in 1828, when white male suffrage had been greatly expanded to almost universal status. These largely uneducated white men, at a time when men and women of color and white women were unilaterally subordinated but abolitionism was on the rise, elected a slaveowner who conducted the First Seminole War, and as president signed the Indian removal act, responsible for the genocide of many native Americans (Watson 2006).

From at least 2015 on, we have seen another populist surge of angry white men, afraid of losing what remains of their power, which women and people of color have supposedly taken away. Though the narrative surrounding Trump’s victory was one of disaffected “regular” Americans who were pushing back against a liberal power elite that was corrupt (“drain the swamp”), in fact his policies predominantly benefitted the wealthy and demonstrated unprecedented levels of corruption, and many of his most passionate supporters are among those further hurt economically by Trump’s mishandling of the 2020 pandemic (Butchireddygari 2020). Yet, echoing British and Italian rhetoric, the demonization of immigrants particularly fuels this populist fantasy; immigrants are said to both proliferate crime and “take our” jobs, despite the evidence that immigration reduces crime (Zatz and Smith 2012) and that most immigrants perform jobs that citizens in the host country do not want (Chomsky 2018).

This tension, if not contradiction, between the theoretical appeal of populism’s supposedly democratic ethos and repeated dissatisfaction with its results in practice has been discussed by many critics of populism who offer varying explanations, as Kim Lane Scheppele has noted (Scheppele, 2019, 5). Some have outright condemned populism in recent years. Michael Herzfeld defines populism as “a performative form of political action in which potentially offensive speech, mannerisms, and attitudes are rendered legitimate as an alternative to establishment values and practices…. that has spread its miasma across the world today” (2019, 134–5). The science fiction writer William Gibson (2017) (whose books served as inspiration for the “Matrix” movies) calls populism “a nightmare state of democracy.” Constitutional theorist Jan-Werner Mueller (2016) says that populists attempt to “hijack” the state apparatus.

Despite those who see populism as democratically hopeful, I believe these accounts accurately reflect the state of populism today. As Scheppele notes, populism is often primarily about populist *leaders*—she focuses particular attention on Hungary’s Viktor Orbán—who are in the majority of cases “simply autocrats,” and patriarchal, racist ones at that (Scheppele, 2019, 3). This view is shared by Norris and Inglehart (2019), who cite authoritarianism as a key characteristic of populists. For Scheppele, what is important is the challenge that such leaders, and their supporters, pose to liberal constitutionalism. Though I agree that is a vital, if not the central, threat to western democracies, including the United States, here I focus on gender and racial equality and the threat posed by the racist sexism of white patriarchy in today’s populism, particularly its United States variant. I will focus particularly on the Black Lives Matter (BLM) movement and Trumpian populism, but given the state of things in the United States with its uncontrolled Covid-19 explosion, resulting in European bans on United States travelers, and a president who endangered the entire world by making false allegations of election fraud and refusing to transfer power, what I will discuss has serious implications for the entire world.

Cas Mudde (2019) argues that populism can only operate through a "host" ideology. In Brexit it was Farage’s “breaking point,” with Britons showing a decided anxiety about immigrants (Ashcroft 2016)–a view remarkably changed by 2018 (Adam and Booth 2018)–a feeling shared by supporters of Italy’s League. In today’s United States, immigration also fueled Trump’s 2016 victory, but our racism is also domestic; Make America Great Again not very subtly suggested that the “greatness” that had supposedly been lost was white male power. There is a swath of America that feels it is losing whatever grip it used to have on power, so it wants that power back, and if it can’t have it, it wants to burn the house down. We witnessed both things at once: a government seeking to undermine the rights of women and racial minorities, exclude immigrants, and punish the poor and working class (including many Trump supporters), and to undermine the legitimacy of the democratic process itself. The house has been on fire for four years, and many so-called populists would now rather see it burned to the ground than lose to a white liberal man and a black woman.

Caren et al. (2020) consider BLM a “social movement” rather than a populist one. Following their lead, I want to argue that what we see now in the twenty-first century requires a conceptual distinction between *populist movement* and *popular protest movement.* Political scientists often link the two. Protests are often considered populist, they are seen to arise from populist movements, by definition: the power of the people against a corrupt government elite is the dictionary definition of populism (Oxford English Dictionary 2002).

But I want to suggest that *popular protest movements* are about trying to get government to change its ways—to change its laws, to change its interpretation of those laws, to change its practices, to honor the results of the electoral process, or even to step down and call for new elections. It asks, urges, demands, that government must act. Even in their strongest protests against injustice, such movements at least implicitly acknowledge the authority of government as the instrument of redress, even as the protests locate government as the agent of such injustice. Feminism, for instance, has a long history of popular protest, from the suffragette parades to the Women’s March on Washington (which again, occurred throughout the world) with its plethora of pink pussy hats. We have marched, we have chanted, we have protested, we have lobbied, we have contributed money, and we have run for office. Yet with a few possible localized exceptions in Spain and Finland (Kantola and Lombardo 2019), feminism has never been considered a *populist* movement.

And although BLM protestors–like twentieth-century suffragettes are seen as engaged in “acts of defiance” (Hooker, 2016, 450), such defiance is not of government per se: it is of brute force that police and others have abrogated to themselves in violation of the rights of citizens. The fact that police are acquitted by internal reviews, or vigilantes are acquitted by jurors, does not mean they embody the government as the representative of the people; it means that government is acting corruptly and dishonestly, as Martin Luther King, Jr so powerfully argued 60 years ago in his “Letter to Birmingham City Jail” (King 1991). BLM protestors are demanding that we recognize this, and are demanding that government change, that police and politicians adhere to the law, including accepting responsibility for the abuse of the force they are granted by the state.

By contrast, populism at this historical moment is exhibited when a group seeks to exercise power itself extra-governmentally. When MAGA protesters march, they carry guns and other weapons, they drive cars loaded with white men in a way meant to threaten, intimidate, and silence—a key characteristic of Arendt’s conception of violence (Arendt 1970). It is not a protest movement, it is a populist one, dedicated to claiming power for itself. Images of right-wing militia being fist bumped by police, or police ignoring armed white militia figures while beating up and arresting peaceful BLM protestors might look as if these actors are working with government, rather than supplanting it. But the reverse is arguably the case: militia are acting outside the law, and police, in approving their actions, stand outside the law themselves. Populist movements, in the deployment of violence, try to act in place of government: government cannot get the job done so they will do it themselves. Power by the people in a literal sense. The attack on the U.S. Capitol was the culmination of such power, as expected and inevitable as it was horrifying.

Violence is a particular hallmark of populism today. In a convex mirror image of Weber, violence is the only extra-governmental action it can engage in. Populist movements cannot issue marriage licenses, or birth certificates, or excavate and pave a new highway. Or rather, in theory, private groups could organize to do these things, but those things will not be recognized outside of the group. So violence is the easiest way for populism to express itself in a way that gets recognized. And as Arendt argues, violence is always illegitimate, always bad, and actually contradicts power. In her typology, BLM exercises power in its peaceful marches, whereas white populists misunderstand what power is and substitute it with force (Arendt 1970). As Ackerman and Bartkowski point out, “numerous studies show that the chances of democratic outcomes from nonviolent movements are vastly higher than for other forms of transitions” (Ackerman and Bartkowski 2017). If populism is about democracy, and if it is to be successful in achieving its goals, then it should eschew violence, not embrace it (see also Gillion, 2016; Gillion, 2020).

Given the philosophy of nonviolent resistance of the civil rights movement (Thoreau 1946; King 1964; Karamchand Gandhi, 1997), I realize that my calling BLM a popular protest movement that should eschew violence may seem to risk the “conceptual trap in romantic historical narratives of black activism (especially the civil rights movement) that recast peaceful acquiescence to loss as a form of democratic exemplarity” that Juliet Hooker warns us against (Hooker, 2016, 448; see Clayton 2018). But that is not what I am arguing for. Of the BLM protests that occurred during the summer of 2020, 93 percent *were* peaceful (Shepherd 2020). But peaceful does not mean passive or acquiescent. Along with Hooker, I firmly reject the “readings of nonviolent protest as acquiescence or sacrifice” (Hooker, 2016, 450). Hooker rightfully criticizes those who claim that “King would be appalled’ by the Black Lives Matter movement” (450). But it is a misreading of King when scholars argue that he would “demand immediate black forgiveness after horrific losses” (Hooker, 2016, 450). King would be angered by the repeated lack of response to African American demands; he said as much in his “Letter from Birmingham City Jail” about the violence expressed against civil rights workers and the resistance of white politicians and voters to civil rights demands (King 1991). King was unhesitating in his criticism of the “white moderate” who is “more devoted to ‘order’ than to justice; who prefers a negative peace which is the absence of tension to a positive peace which is the presence of justice” (King 1991, 75). To say that BLM is a peaceful protest movement is not to urge it to appeasement.

My insistence on rejecting violence might also seem to be critical of the looting and property damage that took place in that remaining seven percent of BLM protests. I am critical of violence against persons when not in active self-defense, and so even the killing of a white nationalist in Portland by a member of so-called antifa is not in keeping with BLM ideals, any more than suffragettes’ using small bombs was justified. The vast majority of suffragettes did not condone violence; they chained themselves to the gates of Parliament and went on hunger strikes, and as a result they were the *victims* of state violence, including police and prison guards, not to mention male citizens hostile to their cause (Teele 2018). Similarly, the vast majority of BLM protesters do not want or advocate violence; they are instead the *victims* of state violence at the hands of police, as well as of populist militia. Those on the side of BLM who engage in violence step outside of that protest framework by failing to honor that nonviolent intention. And they betray the protesters’ intentions, serving as a distraction. Violence within peaceful protests is a bait and switch to undermine the message of the protesters from within and distract attention from what is really going on (such as infiltrators trying to trigger violence to delegitimize the protests).

Yet I sympathize with the view that the tipping over to destruction of property connected with looting—which we should distinguish from violence directed at persons—might be seen as a “form of democratic repair for African-Americans” that makes visible their pain (Hooker, 2016, 464). Hooker points out that since slavery, when blacks had to “steal” themselves in order to attain freedom, African Americans have often had to act “outside the strictly legal” (451). The realism of white power in the United States suggests such views may be naïve about the effects of such actions on achieving the goals of racial equality (Feinberg et al., 2020; but see; Gillion 2020). But more important is the view expressed by Walter Wallace, Sr after the police shooting of his son in Philadelphia in response to looting and particularly an attack on a police officer: “They’re not helping my family; they’re showing disrespect. Stop this violence and chaos. People have businesses. We all got to eat” (Wood and Whelan, 2020). Efforts to rationalize or justify looting also assume a somewhat racist attitude that this property destruction was part of the BLM agenda, which ignores the opportunism of some white criminals who used the protest to hide their actions, such as blowing up ATMs (Rinde 2020), not to mention white agitators hoping to “accelerate” damage to the movement (Byman 2020; see also Bloom 2020).

It also conveniently shifts attention away from where the violence is occurring. It is important to differentiate between destruction of property and the taking of human life and forms of violence against persons exhibited by police and by white vigilantism if we are to acknowledge that it is Trumpian populism that seeks out violence, seeks to engage violent means, and welcomes violence as the method of extra-governmental action. Trump himself seemed to call on the vigilante group the White Boys on national television during the first presidential debate with Joe Biden, seeking to destabilize the very “law and order” platform he claimed to be running on. He similarly urged protesters to violent action against Congress on January 6 to disrupt the certification of election results. His linkage of the need for violence to conspiracy theories about the “deep state” signals, ironically, a rejection of the standards of governmental authority and a move toward authoritarian arbitrary rule. It is this form of violence that I wish to critique as a patriarchal legacy of populism, suggesting that white patriarchy is what motivates this violence.

It is “patriarchal” because, at the most basic level, it is almost exclusively white men who engage this activity. The stories are familiar to anyone reading the news. In Portland, white men driving in a caravan of trucks, with sporting rifles and guns; in Philadelphia, white men “carrying baseball bats, hammers, pipes, and golf clubs” and beating up people of color who did nothing provocative (Griswold 2020). These stories mirrored the actions of police against peaceful BLM protesters. Again in Philadelphia, “police shot rubber bullets and cannisters of tear gas into a crowd of protesters who were kneeling with their arms above their heads, causing them to scatter. The police forced them onto an embankment, trapping them there, and continued tear-gassing them” (Griswold 2020). At that same protest, “a cop pulled down a participant’s mask and pepper-sprayed her in the face. The Bat Boys [a white male vigilante group named for the weapons they carried] in contrast, were treated in a friendly manner” (Griswold 2020). Meanwhile, in Portland, police stood by during a violent clash between a white supremacist group carrying “paintball guns, metal rods, aluminum bats, fireworks, pepper spray, rifles and handguns” and left-wing so-called “antifa” counter-protesters armed with “rocks and fireworks” while previous peaceful BLM marches had been deemed “riots” (Shepherd 2020) and although some women participated in the January 6 attack on the U.S. Capitol, the vast majority were white males.

But a more important reason for calling this patriarchal is that what is distinctive about this violence is that it is “political violence, which is driven by ideology” (Griswold 2020; see also; Noel, 2018; Wynia et al., 2017), the same ideology that fuels this populist moment, namely white male supremacy. Most of the violence that we have seen in response to the social justice protests in the United States in 2020 stems not just from white men but from the expression of a distorted version of white masculinity that resents the perceived loss of position if racial and sexual equality are established. Whether it is police tear gassing peaceful protesters or gun-toting militia, it is certain men fighting the loss of a certain kind of white male privilege. Even when women express violence, such as Patricia McCloskey in St. Louis, they are doing it under the framework of patriarchy; in this case, a wife alongside her husband supposedly defending the family home from an imagined and fantasized violence that the protesters passing their house had no desire to engage in and was never going to happen but for the McCloskey’s own aggressive stance.

Trump is certainly not the cause of these problems in United States. society, though he may have magnified them; he is the symptom. This was arguably true of the Brexit movement as well, which heightened and arguably manipulated through fear-mongering falsities a previously existing hostility to immigrants and persons of color among substantial portions of Great Britain. The current populism gives people permission to express the racism and sexism they had felt compelled to silence during recent years in a backlash formation. And in this animation of populist violence, supporting white supremacists and calling on right wing militias to attack peaceful protestors, he fuels the hope that white patriarchy can be recovered from the losses it has sustained. Yet as Herzfeld rightly notes, this is delusional and only serves the power of the elite (Herzfeld, 2019 135).

Though the change that we need in this country and throughout the world must happen on many different levels, a vital way to fight this deluded populism is through protest movement: continued, relentless, and peaceful. Daniel Gillion (2020) has shown that peaceful protests work to shift the political landscape and affect voting behavior. Populism can sweep people up in a frenzy of anger. But swings in electoral preferences can be readily linked to the success of protest and the unmasking of racial and sexual injustice. The pressure of peaceful protest must continue if we are to have a truly inclusive and democratic world.
